# A Novel Bifunctional Endolytic Alginate Lyase with Variable Alginate-Degrading Modes and Versatile Monosaccharide-Producing Properties

**DOI:** 10.3389/fmicb.2018.00167

**Published:** 2018-02-08

**Authors:** Chune Peng, Qingbin Wang, Danrong Lu, Wenjun Han, Fuchuan Li

**Affiliations:** National Glycoengineering Research Center, Shandong Provincial Key Laboratory of Carbohydrate Chemistry and Glycobiology, State Key Laboratory of Microbial Technology, Shandong University, Jinan, China

**Keywords:** alginate lyase, action mode, *Flammeovirga*, monosaccharide, oligosaccharide-yielding properties

## Abstract

Endo-type alginate lyases usually degrade alginate completely into various size-defined unsaturated oligosaccharide products (≥disaccharides), while exoenzymes primarily produce monosaccharide products including saturated mannuronate (M) and guluronate (G) units and particularly unsaturated Δ units. Recently, two bifunctional alginate lyases have been identified as endolytic but M- and G-producing with variable action modes. However, endolytic Δ-producing alginate lyases remain undiscovered. Herein, a new *Flammeovirga* protein, Aly2, was classified into the polysaccharide lyase 7 superfamily. The recombinant enzyme and its truncated protein showed similar stable biochemical characteristics. Using different sugar chains as testing substrates, we demonstrated that the two enzymes are bifunctional while G-preferring, endolytic whereas monosaccharide-producing. Furthermore, the catalytic module of Aly2 can vary the action modes depending on the terminus type, molecular size, and M/G content of the substrate, thereby yielding different levels of M, G, and Δ units. Notably, the enzymes preferentially produce Δ units when digesting small size-defined oligosaccharide substrates, particularly the smallest substrate (unsaturated tetrasaccharide fractions). Deletion of the non-catalytic region of Aly2 caused weak changes in the action modes and biochemical characteristics. This study provided extended insights into alginate lyase groups with variable action modes for accurate enzyme use.

## Introduction

Alginate is a linear polysaccharide composed of (1-4)-linked β-D-mannuronate (M) and its C5-epimer α-L-guluronate (G) in different sequences. Based on the arrangement of these two uronate residues, the polysaccharide chain comprises three types of inner domains: homo-oligomeric domains of M (M-blocks) and of G (G-blocks) and hetero-oligomeric domains (MG-blocks or GM-blocks) (Pawar and Edgar, 2012). Alginate can be extracted from brown seaweeds or certain bacteria. As the major component of the algal cell wall matrix, alginate accounts for up to 40% of the dry weight of brown seaweeds and represents one large kind of marine biomass (Kim et al., 2011; Abdallah et al., 2016). Acetylated alginate can be synthesized by *Azotobacter* (Gorin and Spencer, 1966) and *Pseudomonas* strains to protect the bacteria from drugs or environmental damage (Rehm and Valla, 1997; Rehm, 2010). Algal alginate and alginate-derived oligosaccharides have attracted increasing interest because of their general utilities in the food, biochemical, and pharmaceutical industries as stabilizing, thickening, or emulsifying reagents (Formo et al., 2014; Abdallah et al., 2016; Chen et al., 2017).

Alginate lyase can cleave the glycosidic bonds of alginate by a β-elimination mechanism, producing oligosaccharide products that contain unsaturated sugar units at the non-reducing end. The newly formed unsaturated residues were derived from G or M units but have lost their original configurations; thus, they are designated as Δ units (Jedrzejas, 2000; Lee and Mooney, 2012; Thomas et al., 2013; Inoue et al., 2016). Therefore, alginate lyases are important tools for the enzymatic preparation of functional oligosaccharides (An et al., 2009; Courtois, 2009; Zhu et al., 2016), the bioconversion of energy that does not compete with food resources (Kovalenko et al., 2011; Martin et al., 2014), and the improvement of antibiotics, assisting their efficiency against biofilm-forming *Pseudomonas* pathogens (Alkawash et al., 2006; Papi et al., 2012). Thus far, numerous alginate lyases have been identified from marine organisms (e.g., algae, mollusks, bacteria, and fungi), terrestrial bacteria, and some viruses (Lee and Mooney, 2012; Pawar and Edgar, 2012; Ertesvag, 2015). In the Carbohydrate-Active enZYmes (CAZy) database, alginate lyases belong to the class of polysaccharide lyases (PL) that are assigned into 24 families based on the sequence similarity of their catalytic modules. Most alginate lyases are classified into the PL-5, 6, 7, 14, 15, 17, and 18 families. Based on their substrate preference, alginate lyases can be classified into three groups, including G-specific lyases (EC4.2.2.11), M-specific lyases (EC4.2.2.3), and bifunctional lyases. Moreover, alginate lyases can be classified as exolytic or endolytic types (Gorin and Spencer, 1966; Sawabe et al., 1997; Wong et al., 2000; Park et al., 2012).

Exo-type alginate lyases can depolymerize alginate by continuously producing monosaccharide products, i.e., M, G, and mainly Δ units, the latter of which can further undergo non-enzymatic conversion into 4-deoxy-L-erythro-5-hexoseulose uronic acid (Kim et al., 2012; Park et al., 2014). Therefore, they are essential for energy bioconversion and carbon cycling in nature. To date, a few exo-type alginate lyases have been identified, such as A1-IV from *Sphingomonas* sp. A1 (Miyake et al., 2003), Atu3025 from *Agrobacterium tumefaciens* (Ochiai et al., 2010), Alg17C from *Saccharophagus degradans* 2-40 (Kim et al., 2012), and AlyA5 from *Zobellia galactanivorans* (Thomas et al., 2013), OalB and OalC from *Vibrio splendidus* (Jagtap et al., 2014). Most of the elucidated alginate lyases are endo-type enzymes, e.g., AlyA1PL7 from *Z. galactanivorans* (Thomas et al., 2013), FlAlyA from *Flavobacterium* sp. strain UMI-01 (Inoue et al., 2014), A1-I, A1-II, and A1-III from *Sphingomonas* sp. strain A1 (Yoon et al., 2000; Miyake et al., 2004; Yamasaki et al., 2005), Aly1 and Aly5 from *Flammeovirga* sp. strain MY04 (Han et al., 2015; Cheng et al., 2017), and FsAlyA from *Flammeovirga* sp. strain NJ-04 (Zhu et al., 2017). Endolytic alginate lyases can be used as tools for the preparation of alginate oligosaccharides, as they efficiently produce unsaturated oligosaccharide products with various degrees of polymerization (DPs), e.g., fractions of unsaturated disaccharide (UDP2), unsaturated trisaccharide (UDP3), unsaturated tetrasaccharide (UDP4), and unsaturated pentasaccharide (UDP5). However, in the final main alginate digests, the molar proportions and structure properties of each size-defined oligosaccharide products are significantly different for each endolytic enzyme due to the enzyme’s unique substrate-degrading patterns, e.g., the substrate preference, the molecular sizes of degradable substrates, and the DPs of the smallest products. Therefore, it is essential to learn the substrate-degrading patterns and the oligosaccharide-yielding properties, i.e., the action modes, of an alginate lyase for accurate enzyme uses.

Most of the elucidated endolytic alginate lyases yield UDP2 or larger fractions as the smallest products and thus produce various size-defined unsaturated oligosaccharide fractions ≥UDP2 as the final main products. Interestingly, in recent studies, two G-preferring bifunctional alginate lyases, a natural enzyme purified from *Alteromonas* sp. strain No. 272 (Iwamoto et al., 2001) and the recombinant enzyme rAly1 of *Flammeovirga* sp. strain MY04 (Cheng et al., 2017), have been identified as endolytic enzymes with similar capacities for producing saturated monosaccharide products of M and G units, albeit at different yield levels. Notably, the two enzymes can produce a series of small size-defined saturated oligosaccharide fractions, e.g., M, M2, and M3 or G, G2, and G3 chains, from the non-reducing end of single or different saturated sugar chains, with parallel size enlargements of the final mail products to substrates, which have been defined as variable action modes (Cheng et al., 2017). Furthermore, we have demonstrated that Aly1 varies its mode of action depending on the terminus type, the molecular size, and the M/G content of substrates. Based on this, we are interested in whether any Δ-producing endolytic alginate lyase exists and in the mechanism of production of the Δ unit, which is important to further understand alginate lyase groups with variable action modes.

In this study, a new putative alginate lyase (Aly2) encoded by the genome of *Flammeovirga* sp. strain MY04 was cloned to obtain the whole protein and a gene-truncated version containing the protein without the non-catalytic region (NCR). Furthermore, the biochemical characteristics, substrate-degrading patterns, and oligosaccharide-yielding properties of the two enzymes were compared.

## Experimental Procedures

### Materials and Strains

Prime STAR^TM^ HS DNA polymerase, restriction endonuclease, T4 DNA ligase, and other genetic engineering enzymes were purchased from TaKaRa Inc. (Dalian, China). Sodium alginate (alginic acid sodium salt from brown algae, medium viscosity) was purchased from Sigma-Aldrich Co., Ltd., United States. M- and G-enriched disaccharide, trisaccharide, tetrasaccharide, pentasaccharide, and hexasaccharide (>95% reported purity) were purchased from Qingdao BZ Oligo Biotech Co., Ltd. (Qingdao, China). UDP3, UDP4, and UDP5 were prepared by the digestion of alginate using alginate lyase rAly5. Polyguluronate (PG) and polymannuronate (PM) (purity of approximately 95%) were prepared from sodium alginate according to Haug et al. (1967).

The marine bacterium *Flammeovirga* sp. strain MY04 (CGMCC No. 2777) was cultured in a medium (pH 7.0) containing (w/v) 0.40% tryptone, 0.25% yeast extract, and 3.0% NaCl at 30°C. *Escherichia coli* strains were cultured at 37°C in Luria-Bertani (LB) broth or on LB broth agar (LB broth supplemented with 1.5% agar) with kanamycin (50 μg/ml).

### Sequence Analysis of Gene and Protein of Alginate Lyase

Promoter motifs of the 5′-flanking DNA region upstream of the open reading frame (ORF) were identified using Primer Premier version 5.0 (PREMIER Biosoft International, Palo Alto, CA, United States) and the Promoter 2.0 Prediction Server. The GC content (GC%) of the ORF and the sequence alignment were calculated using Bio-Edit version 7.2.5 (Hall, 1999). An online similarity search for the protein sequence was performed using the BLASTp algorithm on NCBI^1^. The secretion signal peptide and its type were identified using the SignalP 4.0 server and the LipoP 1.0 server^2^. The molecular mass of the protein was estimated using the peptide mass tool on the ExPASy server of the Swiss Institute of Bioinformatics^3^. Protein modules and domains were identified using the Simple Modular Architecture Research Tool (SMART)^4^. Multiple sequence alignments and phylogenetic analyses were performed using MEGA version 6.01 (Kumar et al., 1994).

### Heterologous Expression of rAly2 and the Truncated Protein

The genome of *Flammeovirga* sp. strain MY04 isolated from sea mud encodes at least five putative alginate lyases. In this study, we expressed the ORF2544, Aly2 (GenBank accession number ANQ49913.1). To express the whole protein (rAly2) and its NCR-truncated protein (rT282N) in *E. coli* strains, the full-length gene (*aly2*) without the signal peptide sequence was amplified using high fidelity Prime STAR^TM^ HS DNA polymerases (TaKaRa Inc., Dalian, China) and the primer pair 30aAly2-F (5′-CGGATCCCAACAACCTATCGTTATTGTAAAC-3′) and 30aAly2-R (5′-GCTCGAGTTTTATTTGGTGTATAAGTGGTTTTTAAC-3′). The truncated gene was amplified using the primer pair T282N-F (5′-CGGATCCGTGATTTTAAATCAAGAACTAGATG-3′) and T282N-R (5′-GCTCGAGTTTTATTTGGTGTATAAGTGGTTTTTAAC-3′). Primer pairs with restriction enzyme sites *Bam*HI–*Xho*I (underlined) were designed according to the insertion site sequences of the expression plasmids, pET-30a (+) (Novagen), with a His_6_ tag at the C-terminus of the recombinant protein of rAly2 or rT282N. The recombinant plasmid (pE30a-rAly2 or pE30a-T282N) was amplified in *E. coli* DH5α cells and was then individually transformed into *E. coli* BL21(DE3) cells for protein expression. The integrity of the nucleotide sequences of all the constructed plasmids were confirmed by DNA sequencing.

*Escherichia coli* cells harboring a recombinant plasmid were initially cultured in LB broth. When cell density reached an OD_600_ of 0.8–1.0, the broth was supplemented with the inducer isopropyl 1-thio-β-D-galactopyranoside at a final concentration of 0.05 mM to start the expression of the target proteins. After continuous cultivation for an additional 24 h at 16°C, the cells were harvested by centrifugation at 6,000 × *g* for 15 min. The pellet was resuspended twice using ice-cold buffer A [50 mM Tris–HCl, 150 mM NaCl (pH 8.0)] and disrupted by sonication (60 repetitions, 5 s) in an ice-cold environment. After centrifugation at 15,000 × *g* for 30 min, the supernatant was collected for further purification of the soluble target proteins.

### Purification of the Two Recombinant Proteins rAly2 and rT282N

To purify the target proteins, the supernatant containing each soluble enzyme was loaded onto a column packed with nickel-Sepharose^TM^ 6 Fast Flow resin (GE Healthcare, United States), washed with buffer A containing 20 mM imidazole to remove impurities, and finally eluted with a gradient concentration of imidazole, ranging from 50 to 250 mM (Han et al., 2015; Cheng et al., 2017). Fractions were analyzed by sodium dodecyl sulfate polyacrylamide gel electrophoresis (SDS-PAGE) according to the method reported by Sambrook and Russell (2006). The proteins were visualized by staining using Coomassie Brilliant Blue R-250. The recombinant protein-containing fraction was pooled, and the protein concentration was determined by the bicinchoninic acid method.

### Biochemical Characteristics of the Recombinant Proteins rAly2 and rT282N

To determine the optimal pH for the enzyme activity, sodium alginate (1 mg/ml) was reacted with 1 μg of rAly2 or 1 μg of rT282N in buffers with different pH values, including a final concentration of 50 mM NaAc-HAc buffer (pH 5.0–6.0), NaH_2_PO_4_–Na_2_HPO_4_ buffer (pH 6.0–8.0), or Tris–HCl buffer (pH 7.0–10.0) in a total volume of 60 μl at 40°C for 2 h. After the optimum pH was determined, the effects of temperature on the activities of the two enzymes were tested in 50 mM NaAc-HAc buffer (pH 6.0) at temperatures from 0 to 100°C for 2 h. The effects of metal ions/chelating reagents (1 and 10 mM) on the activities of the two enzymes were further investigated at the optimum pH and temperature described above. To determine the thermostability, the enzyme in 50 mM NaAc-HAc buffer (pH 6.0) was preincubated for 0–24 h at a temperature from 0 to 90°C, and the residual alginate-degrading activity was determined at 40°C. All reactions were performed in triplicate, and after each treatment, the enzyme activity was estimated by measuring the absorbance at 235 nm. All reactions were performed in triplicate. Error bars represent means of triplicates, and indicated as “means ± SD”.

### Optimum Assay Conditions for rAly2 and rT282N

The enzyme activities were measured according to the method provided by Gacesa (1992). Briefly, rAly2 or rT282N (2 μg) was individually added to 1 mg/ml sodium alginate in 50 mM NaAc-HAc buffer (pH 6.0) in a total volume of 1 ml. The reaction mixture was incubated at 40°C. At various time intervals (up to 10 min), aliquots of 100 μl were withdrawn in duplicate, boiled for 10 min, and then ice-cooled for 10 min. After centrifugation at 15,000 × *g* for 10 min, the supernatant was collected, diluted to 200 μl, and analyzed using the absorbance at 235 nm. One unit (U) was defined as the amount of enzyme required to increase the absorbance at 235 nm by 0.1/min. The *K*_m_ values for rAly2 and rT282N toward sodium alginate, PM and PG were determined by non-linear analysis based on the initial rates determined with 0.25–4 mg/ml of each substrate at 40°C.

### Gel Filtration Chromatography

Samples digested by rAly2 or rT282N were analyzed by gel filtration chromatography on a Superdex^TM^ Peptide 10/300 GL column. The mobile phase was 0.20 M NH_4_HCO_3_ at a flow rate of 0.4 ml/min, and the eluted fractions were monitored at 235 nm using a UV detector. Online monitoring and data analysis (e.g., molar ratio determination) were performed using the software LC solution version 1.25.

### Polysaccharide-Degrading Pattern of rAly2 and rT282N

To determine the substrate-degrading pattern of the two alginate lyases, the digests of sodium alginate (1 mg/ml) obtained by using rAly2 (1 μg) or rT282N (1 μg) were traced at 40°C for 72 h. Aliquots of the reaction products (20 μg) were removed for time course experiments to analyze gel filtration chromatography by monitoring at 235 nm. Sodium alginate, PM, and PG were exhaustively digested by rAly2 and rT282N, separately, and the final products were loaded onto a Superdex^TM^ Peptide 10/300 GL column as described above.

To further determine the molecular weights and structural properties of the final oligosaccharide products, enzymatic degradation was individually performed on 10 ml of 1 mg/ml sodium alginate samples with excessive amounts of rAly2 or rT282N at optimal conditions for 72 h. The reaction mixtures were heated in boiling water for 10 min and subsequently cooled to 4°C. The samples were centrifuged at 15,000 × *g* for 30 min, and then, the supernatant was loaded onto a pre-equilibrated Superdex^TM^ Peptide 10/300 GL column at room temperature. Unsaturated oligosaccharide samples were collected with online monitoring at 235 nm and freeze-dried repeatedly to remove NH_4_HCO_3_ for further identification.

The UDP2, UDP3, and UDP4 fractions were purified from the final alginate digests by each enzyme and further identified by MS on an ion trap TOF hybrid mass spectrometer (LCMS-IT-TOF, Shimadzu, Japan). The electrospray ionization MS analysis was set in the negative ion mode using the following parameters: a source voltage of 3.6 kV, a nebulizer nitrogen gas flow rate of 1.5 l/min, a heat block and curved desolvation line temperature of 200°C, and a detector voltage of 1.8 kV. The mass acquisition range was set at 0–1000.

### Structural Identity of the Final Products of rAly2 and rT282N

To determine the structures of each size-defined final oligosaccharide product, the signals of UDP2 and UDP3 fractions were assigned from the one-dimensional proton NMR spectra referenced at 30°C. Each purified oligosaccharide fraction (2 mg) was dissolved in 0.5 ml of D_2_O in 5-mm NMR tubes. The spectra were recorded on a JNM-ECP600 (JEOL, Japan) apparatus set at 600 MHz.

To further determine the number of components in the UDP3 fraction, the purified UDP3 fraction from alginate was analyzed by anion-exchange HPLC on a YMC-Pack PA-G column eluted with a linear gradient from 16 to 460 mM NaH_2_PO_4_ over a 60-min period and monitored using a UV detector at 235 nm.

### Analysis of the Oligosaccharide- Degrading Properties of rAly2

To determine the unsaturated oligosaccharide-degrading properties of rAly2 and rT282N, a total of 10 mg of sodium alginate was partially digested by rAly5 at 40°C as described previously. The resulting UDP2, UDP3, UDP4, and UDP5 fractions were fractionated using gel filtration on a Superdex^TM^ Peptide 10/300 GL column. The oligosaccharide fraction (∼20 μg) was digested with exhaustive enzyme in a total volume of 20 μl at 40°C, and the digests were analyzed by HPLC using a Superdex^TM^ peptide 10/300 GL column with monitoring at 235 nm.

To further determine the saturated oligosaccharide-degrading properties of rAly2, saturated oligosaccharides (M2-M6, G2-G6) were digested by the enzyme under optimal conditions for 12 h, and HPLC analyses were performed as described above.

## Results

### Alginate Lyase Gene and Protein Sequence

**FIGURE 1:**
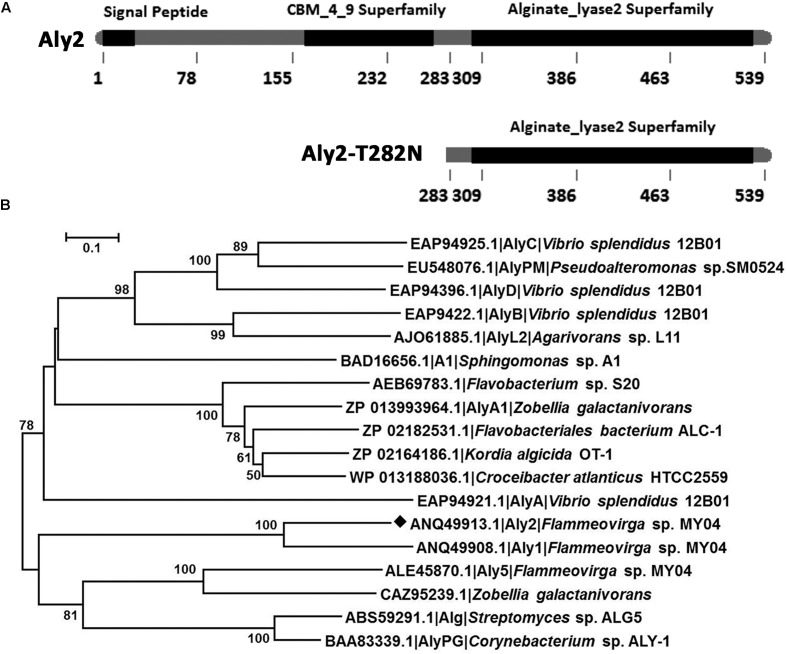
Module organization type of the alginate lyase Aly2 from *Flammeovirga* sp. strain MY04. **(A)** Module organization of the alginate lyase Aly2. It contains a putative N-terminal signal peptide (Met^1^-Ala^26^), a hypothetical carbohydrate-binding module of the CBM_4_9 superfamily (Leu^165^-Thr^270^) and a putative catalytic module of alginate_lyase2 superfamily (Leu^302^-Ile^529^). The full-length protein was expressed to yield the recombinant protein rAly2 and the non-catalytic region (NCR) (Met^1^-Val^282^) was gene-deleted to obtain the truncated protein rT282N. **(B)** Phylogenic tree of alginate lyases based on protein sequence alignments. The tree was generated using the neighbor-joining method and MEGA version 5.05 software. The numbers on the branches indicate the bootstrap confidence values from 1,000 replicates. The bar is equal to the distance corresponding to one amino acid substitution per 10 amino acids.

The putative alginate lyase gene *aly2* (GenBank^TM^ accession number ANQ49913.1) is 1,620 bp in length and has a GC content of 36.1%. The predicted full-length alginate lyase (Aly2) is composed of 539 amino acid residues with a theoretical protein molecular weight of 60.58 kDa. The predicted isoelectric point (p*I*) is 9.14. SignalP 4.0 and LipoP 1.0 analyses indicated that Aly2 contains a type I signal peptide composed of 26 amino acid residues at its N-terminus. CAZy database^5^ and SMART analyses showed that the Aly2 protein contains a CBM_4_9 superfamily module (Leu^165^-Thr^270^) and an alginate_lyase2 superfamily (Alg2) module (Leu^302^-Ile^529^) (**Figure 1A**). BLASTp searches showed that among the elucidated alginate lyases, the full-length sequence shares the highest sequence identity (46%) with Aly1, a bifunctional endolytic alginate lyase from the same bacteria *Flammeovirga* sp. strain MY04 (Cheng et al., 2017), followed by a PG-specific lyase (30%) from *Corynebacterium* sp. strain ALY-1 (Matsubara et al., 1998). Phylogenic analysis suggested that Aly2 is a novel alginate lyase from the genus *Flammeovirga* and belongs to a PL7 subclass to be defined (**Figure 1B**). Protein sequence alignment showed that the alginate_lyase2 superfamily (Alg2) module contains the conserved motifs (Gln^494^-Ile^495^-His^496^, QIH), RXEXR, and YXKAGXYXQ (X, any amino acid), which are conservative in elucidated PL7 alginate lyases (**Supplementary Figure S1**) to constitute the active center and are crucial for substrate recognition and catalysis (Yamasaki et al., 2004; Kawamoto et al., 2006).

### Heterologous Overexpression of Recombinant Alginate Lyases

The full-length gene and the truncated gene of the alginate lyase were amplified directly from the genomic DNA of *Flammeovirga* sp. strain MY04, and the two PCR products were individually cloned into the pET-30a (+) vector following a T7 promoter. In each recombinant protein expression vector, a His_6_ tag was added at the C-terminus of the recombinant protein. SDS-PAGE analysis indicated that *E. coli* BL21(DE3) cells harboring the pE30-Aly2 or pE30-T282N plasmids could each form soluble products.

**FIGURE 2:**
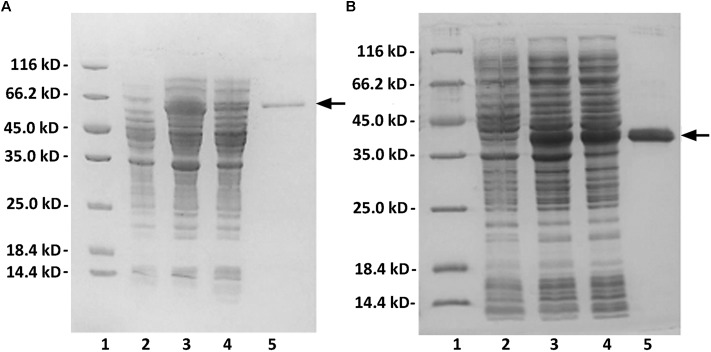
Purification of recombinant rAly2 **(A)** and rT282N **(B)** from *E. coli* by Ni^2+^ chelation chromatography. Enzyme purity following each fractionation step was assessed by SDS-PAGE using 13.2% (w/v) polyacrylamide gels, followed by staining with Coomassie Brilliant Blue. Lane 1, unstained protein molecular weight marker SM 0431; lane 2, induced cell lysate of *E. coli* strains harboring the control plasmid pET-30a (+); lane 3, induced cell lysate of *E. coli* cells containing plasmid of pE30-Aly2 **(A)** or pE30-T282N **(B)**; lane 4, supernatant fluid of the induced cell lysate; lane 5, rAly2 **(A)** or rT282N **(B)** protein purified from supernatant.

The crude enzymes were extracted from the cultures of host cells by sonication and centrifugation. The recombinant enzymes were further purified by Ni-NTA affinity chromatography. As shown in **Figure 2**, SDS-PAGE showed that the rAly2 (**Figure 2A**) and rT282N (**Figure 2B**) proteins could be eluted from the Ni-NTA column using a gradient of imidazole concentrations ranging from 50 to 250 mM. The purified proteins showed molecular masses consistent with the theoretical values and purities >99%. The protein concentration of rAly2 was 2.7 mg/l, while that of rT282N was 13.5 mg/l (**Table 1**), and therefore, five times the yield of rT282N compared to rAly2 was achieved (**Table 1**).

**Table 1:** Purification of the alginate lyases from *E. coli* BL21 (DE3).

	rAly2				rT282N			
		
	Total protein (mg)	Total activity (U)	Specific activity (U/mg)	Yield (%)	Total protein (mg)	Total activity (U)	Specific activity (U/mg)	Yield (%)
Crude protein	20.2	30611.1	515.4	100	70.5	96112.6	1363.3	100
Elution from Ni^2+^ column	2.7	5469.6	2025.8	17.7	13.5	39426.7	2920.5	41.0


### Biochemical Characteristics of rAly2 and rT282N

The protein rAly2 and its NCR-truncated protein rT282N can degrade alginate and blocks of PM and PG, producing oligosaccharide products with an absorbance at 235 nm, which is a typical characteristic of unsaturated oligosaccharides. The results indicated that the protein Aly2 is an alginate lyase that degrades alginate and associated substrates by a β-elimination mechanism.

**FIGURE 3:**
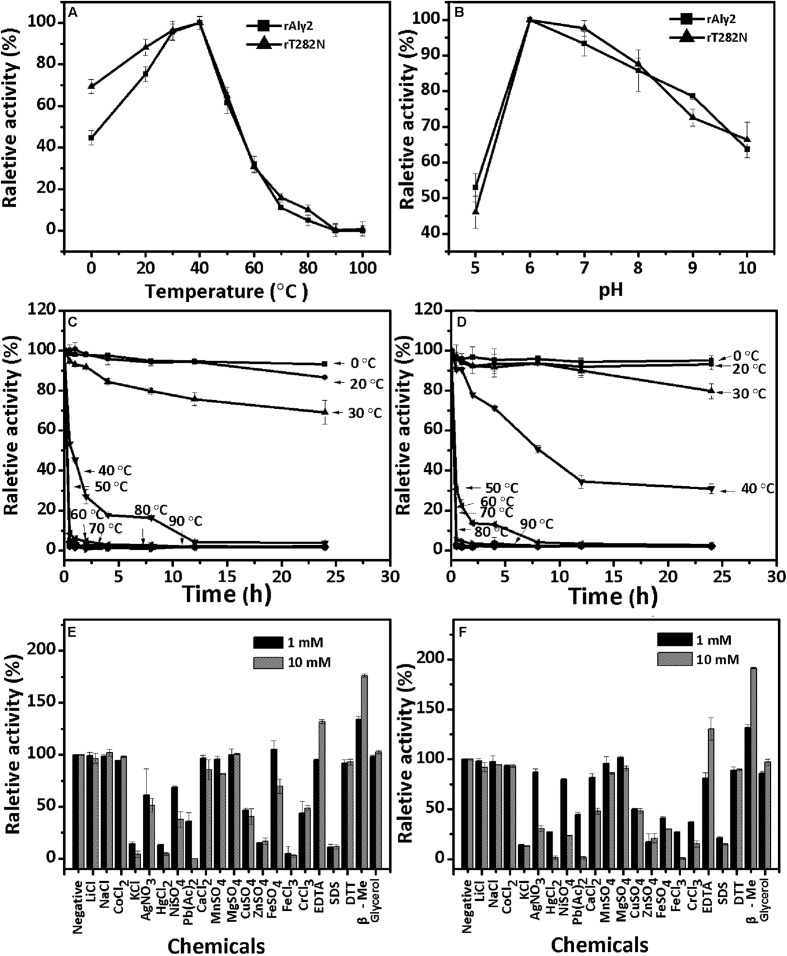
Biochemical characteristics of the recombinant enzyme rAly2 and rT282N. **(A)** Effects of temperature. The enzyme activities of rAly2 and rT282N were each measured using sodium alginate as substrate in the 50 mM NaAc-HAc buffer (pH 6.0) at different temperatures for 2 h. Data are shown as the percentage of the activity of that obtained at 40°C (100%) for rAly2 and rT282N. **(B)** Effects of pH values. The enzyme activities of rAly2 and rT282N against sodium alginate were individually measured in buffers with varying pH values from 5 to 10 at 40°C for 2 h. Data are shown as the percentage of the activity of that obtained in the 50 mM NaAc-HAc buffer at pH 6.0 (100%). **(C,D)** Thermostability of rAly2 and rT282N. The enzyme in 50 mM NaAc-HAc buffer (pH 6.0) was preincubated for 0 to 24 h under temperatures ranging from 0 to 90°C, and the residual activity against sodium alginate was estimated at 40°C. Data are shown as the activity relative to that of untreated rAly2 and rT282N. The data showed that the enzymes are stable at 0–30°C. **(E,F)** Effects of metal ions. The enzyme activities of rAly2 and rT282N against sodium alginate were individually measured in the NaAc-HAc buffer (pH 6.0) containing a 1 mM or 10 mM concentration of various metal ions at 40°C for 2 h. Data are shown as the percentage of the activity of that obtained in the buffer without tested metal ions. Error bars represent means of triplicates ± SD.

The enzyme rAly2 and its truncation protein rT282N showed an optimum temperature of 40°C when sodium alginate was used as the substrate (**Figures 3A,B**). Thermostability assays further showed that their activities are not stable at 40°C if compared to the activities at 0–30°C (**Figures 3C,D**). The enzyme rAly2 retained less than 30% activity after 2 h of preincubation at 40°C for 2 h (**Figure 3C**). The enzyme rT282N showed greater temperature stability and retained greater than 50% activity when it was preincubated at 40°C for 8 h (**Figure 3D**). The optimal pH determined at 40°C was 6.0 in 50 mM NaAc-HAc buffer.

To determine the effects of metal ions on the activity of rAly2, various metal ions were added to the basic reaction buffer (50 mM NaAc-HAc, pH 6.0). As shown in **Figure 3E**, the alginate-degrading activity of rAly2 was slightly affected by the basic alkali Li^+^, Na^+^, K^+^, Ca^2+^, Mg^2+^, and Mn^2+^, slightly inhibited by Co^2+^, Ni^2+^, Fe^2+^, Cu^2+^, and Cr^3+^, and strongly inhibited by Ag^+^, Hg^2+^, Pb^2+^, Fe^3+^, Zn^2+^, and SDS at a concentration of 1.0 and 10.0 mM. Metal ions were not necessary for the activity of rAly2 in this study. The alginate-degrading activity of rAly2 was slightly improved by 10.0 mM glycerol. The presence of the reducing agent 10 mM β-mercaptoethanol (β-Me) increased the enzymatic activity by more than 180%, suggesting that breaking disulfide linkages may increase the enzymatic activity of Aly2. The effects of metal ions and reducing regents on the enzyme activities of rT282N have also been investigated, and the results were similar to those for rAly2 (i.e., metal ions did not improve the activity and the reducing agent β-Me did improve the activity significantly; **Figure 3F**).

Under the optimal conditions of 50 mM NaAc-HAc (pH 6.0) at 40°C, the specific activities of rAly2 and rT282N were measured as described in the Section “Experimental Procedures.” The results (**Table 2**) showed that the lyase rAly2 can degrade both PM and PG. The specific activities of rAly2 against sodium alginate, PM and PG were 2025.8, 1324.9, and 3672.6 U/mg of protein, respectively. The specific activities of rT282N against sodium alginate, PM and PG were 2920.5, 1876.3, and 5141.5 U/mg of protein, higher than those of rAly2. These results indicated that Aly2 is a bifunctional alginate lyase and prefers G to M, as is the case for its truncated protein rT282N. Furthermore, both rAly2 and rT282N have the highest affinity to sodium alginate, shown by their low *K*_m_ values toward sodium alginate (**Table 2**), and their kinetic plots were shown as **Supplementary Figures S2**, **S3**.

**Table 2:** Specific activities and kinetic parameters of rAly2 and rT282N toward sodium alginate, poly M, and poly G.

	rAly2			rT282N		
		
	Sodium alginate	PM	PG	Sodium alginate	PM	PG
Specific activity (U/mg)	2025.8	1324.9	3672.6	2920.5	1876.3	5141.5
*K*_m_ (mg/ml)	0.135	2.845	0.706	0.158	0.392	1.224


### Degradation Pattern of Polysaccharides by rAly2 and rT282N

**FIGURE 4:**
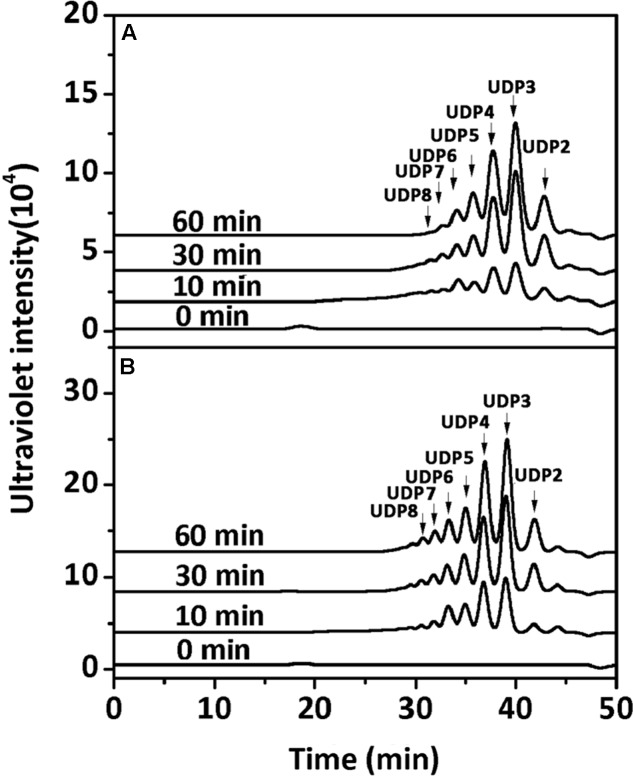
Degradation patterns of rAly2 **(A)** and rT282N **(B)** toward sodium alginate. Sodium alginate (1 mg/ml) was treated with each enzyme (1 μg) at 40°C. A 20 μl aliquot was taken at different time points for gel filtration analysis as described in the Section “Experimental Procedures.” The elution positions of the unsaturated oligosaccharide product fractions with different degrees of polymerization are indicated by arrows: UDP2, unsaturated disaccharide; UDP3, unsaturated trisaccharide, … UDP8, unsaturated octasaccharide.

To determine the degradation patterns of rAly2 (**Figure 4A**) and rT282N (**Figure 4B**), the degradation of sodium alginate (1 mg/ml) by rAly2 and rT282N (1 μg) was individually monitored at 40°C. The reaction times intervals used were 0, 10, 30, and 60 min. The products were loaded onto a Superdex^TM^ Peptide 10/300 GL column and were monitored using the absorbance at 235 nm. The two enzymes, rAly2 and rT282N, initially produced higher molecular mass oligosaccharides and then smaller oligomers with strong absorbance at 235 nm, suggesting that the catalytic module Alg2 (alginate_lyase2 superfamily) of Aly2 degrade sodium alginate polysaccharides using endolytic pattern (**Figure 4**).

**FIGURE 5:**
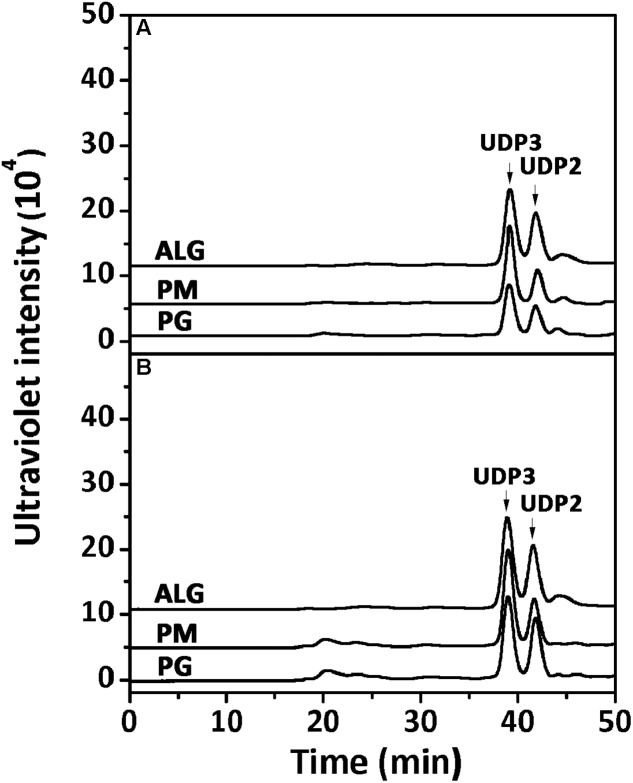
Analysis of the final digests of sodium alginate, poly M, and poly G by rAly2 **(A)** and rT282N **(B)**. Twenty micrograms of sodium alginate (ALG), PM, or PG was exhaustively digested with rAly2 **(A)** and rT282N **(B)**, separately, and then gel filtrated using a Superdex^TM^ Peptide column as described in the Section “Experimental Procedures.” The elution positions of the unsaturated oligosaccharide product fractions with different degrees of polymerization are indicated by arrows: UDP2, unsaturated disaccharide; UDP3, unsaturated trisaccharide.

Furthermore, the polysaccharide substrates, i.e., sodium alginate, PM, and PG, were individually exhaustively digested by rAly2 (**Figure 5A**) or rT282N (**Figure 5B**) at 40°C for 72 h to determine their final oligosaccharide products. The resulting oligosaccharide products were analyzed and assigned by gel filtration analysis. As shown in **Figure 5**, compared to the retention times reported previously, UDP3 and UDP2 fractions were the final main products of each tested substrate, the quantity of UDP4 faction is too small to visible. Based on the peak area integration, the molar ratio between the final UDP2 and UDP3 product fractions was determined to be ∼1:2.1 when the two enzymes degraded the designated alginate substrate.

### Identification of the Final Main Oligosaccharide Products from Sodium Alginate

**FIGURE 6:**
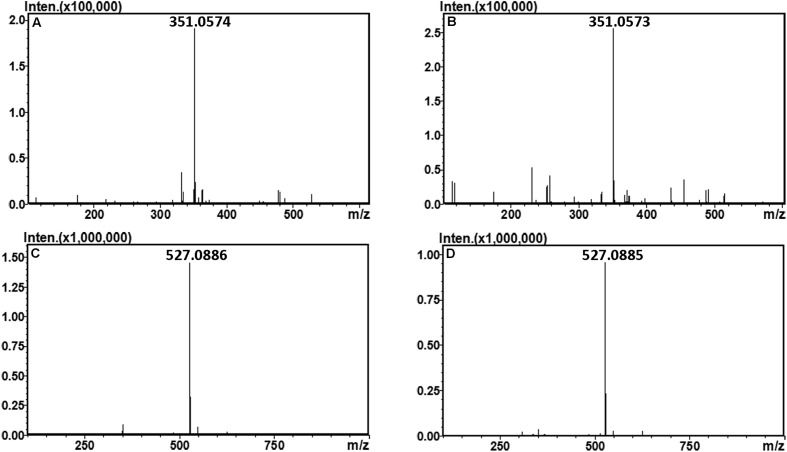
Time-of-flight mass spectra of the final oligosaccharide products of sodium alginate digested by rAly2 and rT282N. The main final products obtained from sodium alginate digested each of enzymes were identified by electrospray ionization MS on anion trap TOF hybrid mass spectrometer as described under Section “Experimental Procedures.” The products are indicated as: UDP2 produced by rAly2 **(A)** and rT282N **(B)**; UDP3 produced by rAly2 **(C)**, and rT282N **(D)**.

The final oligosaccharide products in the sodium alginate (10 mg) digestion by rAly2 or rT282N were separated by size exclusion chromatography as described in the Section “Experimental Procedures.” The two obtained oligosaccharide fractions showed molecular masses of 351.05 (**Figures 6A,B**) and 527.08 (**Figures 6C,D**) in MS analyses, respectively. The results demonstrated that UDP2 and UDP3 fragments constitute the final main oligosaccharide products of rAly2, as well as of rT282N.

**FIGURE 7:**
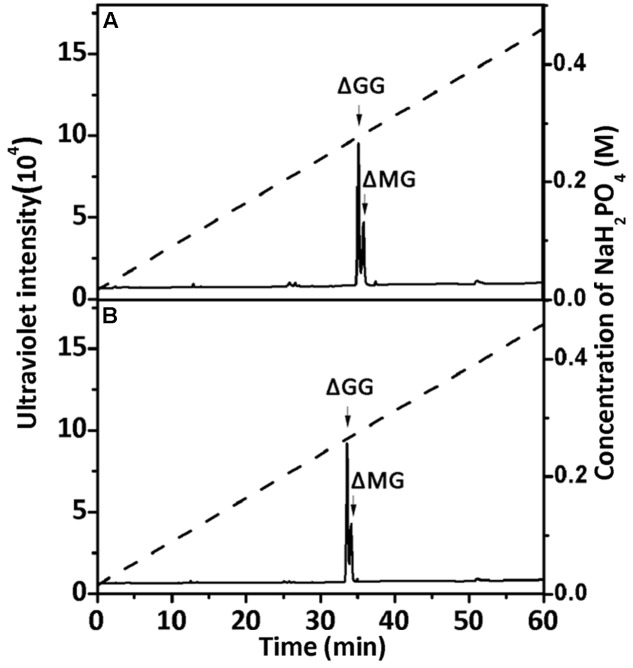
Ion-exchanging HPLC analysis of the unsaturated trisaccharide fraction produced by rAly2 **(A)** and rT282N **(B)**. The unsaturated trisaccharide fraction was analyzed by HPLC on a YMC-Pack PA-G column using a NaH_2_PO_4_ linear gradient from 16 to 460 mM NaH_2_PO_4_ (indicated by the dashed line) over a 60-min period and monitored using a UV detector.

To confirm the properties of the final UDP2, UDP3, and UDP4 product fractions, each of them were analyzed by ^1^H-NMR. After the final UDP3 fractions (20 μg) were loaded on a YMC-Pack PA-G column (**Figure 7**), two different components were separated, with different molar ratios for rAly2 (**Figure 7A**) and rT282N (**Figure 7B**).

**FIGURE 8:**
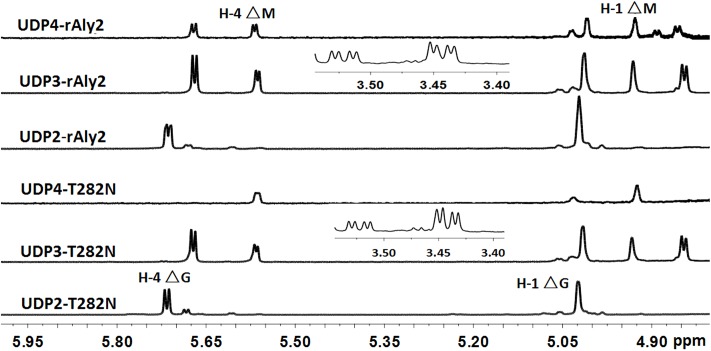
^1^H NMR (600 MHz, 28°C) spectra of the final main oligosaccharide product fractions of sodium alginate by rAly2 and rT282N. Each size-defined oligosaccharide product fractions produced by the two enzymes were individually purified using a Superdex^TM^ peptide 10/300 GL column, monitored at 235 nm. H-4 Δ doublet at 5.71 or 5.67 ppm indicated that the neighbor to Δ is a G residue, meaning that ΔG constitutes the first two sugar residues at the nr ends. The chemical shift of the H-4 Δ signal at 5.56 ppm means that ΔM constitutes the first two sugar residues at the nr ends. As shown in the figure, the intensity of the H-4 ΔG signal is higher than that of H-4 ΔM for trisaccharide products, the intensity of the H-4 ΔM signal is higher than that of H-4 ΔG for UDP4 fraction, demonstrating that the final UDP4 product fractions contain ΔM ends at greater proportions than ΔG ends.

The ^1^H-NMR chemical shifts were assigned according to previously published data for alginate oligosaccharides (Heyraud et al., 1996; Zhang et al., 2006; Lundqvist et al., 2012; Han et al., 2015). The structure of the reducing end sugar can be easily identified by the characteristic chemical shift of its anomeric proton signal. The chemical shifts of the protons of unsaturated non-reducing ends are dependent on the nature of the nearest sugar residue, as identified from the shift of the H-4 signal of Δ. Based on the presence of a strong H-4 ΔG signal at 5.72 ppm and a weak H-4 ΔM signal at 5.61 ppm, the UDP2 fractions from rAly2 were predicted to contain ΔG and ΔM disaccharide units at a molar ratio of ∼10.53:1. In contrast, the UDP2 fraction produced by rT282N has a molar ratio of ∼13.91:1 (**Figure 8**). Thus, ΔM units contributed to a mass concentration of 8.7% of the UDP2 products for rAly2 and 6.7% (w/w) for rT282N, demonstrating that rAly2 and rT282N mainly yield ΔG as a disaccharide product when degrading the designated alginate substrate. The UDP3 and UDP4 fraction showed a H-4 (Δ) doublet at 5.67 ppm, indicating that the neighbor to the Δ unit is a G residue, while the chemical shift of the H-4 signal appearing at 5.56 ppm means that its neighbor is a M residue. As shown in **Figure 8**, the two signals at 3.44 and 3.53 ppm in the UDP3 fraction indicated the existence of H-2 of G at the reducing end, neighboring G and M, respectively (Han et al., 2015). UDP3 fractions from rAly2 and rT282N showed a high content of ΔG in their non-reducing ends, i.e., the ratios of ΔG to ΔM units were determined to be ∼1.49:1 and 1.65:1, respectively. Thus, the ΔGG and ΔMG units were determined to have a molar ratio of ∼1.49:1 for rAly2 and 1.65:1 for rT282N individually, which are consistent with the ratios determined in the anion exchange HPLC analysis (**Figure 7**). Furthermore, the intensity of the H-4 ΔG signal is much higher than that of H-4 ΔM for UDP2 and UDP3, but for larger final UDP4 fractions contain ΔM ends at greater proportions than ΔG ends in their non-reducing ends compared to small final UDP2 and UDP3 final fractions (**Figure 8**). Therefore, although Aly2 yielded large products containing ΔM ends, Aly2 preferred to produce small oligosaccharides in which the non-reducing ends primarily contained ΔG units. As a result, we speculate that the activity on PG is higher than that on PM, which is consistent with the results shown in **Table 2**. The results demonstrated that Aly2 is a bifunctional alginate lyase and prefers G to M. Moreover, the catalytic module Alg2 of the alginate lyase Aly2 plays a key role in determining the polysaccharide-degrading pattern and the oligosaccharide-yielding properties.

### Pattern of Degradation of Oligosaccharide Substrates by rAly2 and rT282

**FIGURE 9:**
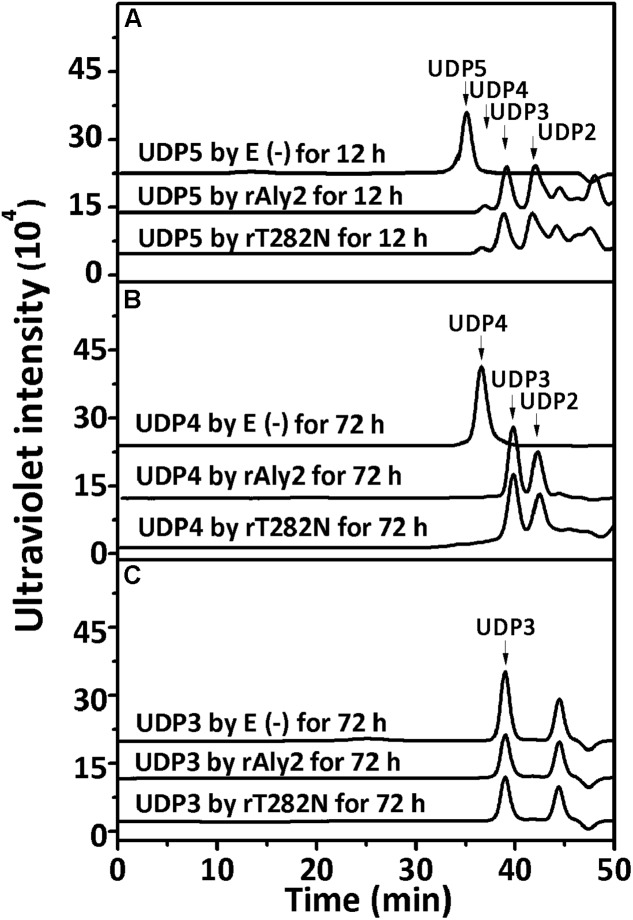
Degradation patterns of rAly2 and its truncated protein rT282N toward unsaturated fractions of pentasaccharide **(A)**, tetrasaccharide **(B)**, and trisaccharide **(C)**. Partial degradation of ∼20 μg UDP5 fractions **(A)** and exhaustive degradation of ∼20 μg UDP4 **(B)**, or UDP3 **(C)** fractions by rAly2 and rT282N at 40°C individually. E (–), without the enzyme; E (+), with the exhaustive enzyme. HPLC analyses were performed using a Superdex^TM^ peptide 10/300 GL column monitored at 235 nm.

To determine and compare the degradation patterns of the two enzymes toward oligosaccharide substrates, sodium alginate was initially partially digested by rAly5 as previously described (Han et al., 2015). Subsequently, the intermediate product fractions of UDP2, UDP3, UDP4, and UDP5 were collected and pooled through gel filtration using a Superdex^TM^ Peptide 10/300 GL column and finally characterized using MS analysis. After further enzymatic reactions with rAly2 or rT282N, the final digests of the UDP4 and UDP5 fractions were analyzed by gel filtration HPLC as described above. These results showed that rAly2 and rT282N could not degrade UDP2 fractions (data not shown) or digest UDP3 fractions to produce any Δ and UDP2 products (**Figure 9C**). The two enzymes could completely degrade the UDP4 fraction to yield Δ and UDP3 or two UDP2 product fractions (**Figure 9B**), with a molar ratio of 2.8:1 between the two different metabolic pathways. When reacted with the UDP5 fraction, rAly2 and rT282N initially yielded products of Δ and UDP4 or UDP2 and UDP3 (**Figure 9A**) and finally converted them into UDP2 and UDP3 products (data not shown). Thus, the smallest substrate for Aly2 is the UDP4 fraction, and the minimal product of Aly2 is the unsaturated monosaccharide Δ. The results also demonstrated that Aly2 has variable alginate-degrading modes when degrading the UDP4 fractions, thereby producing a series of small size-defined unsaturated oligosaccharide products, i.e., Δ, UDP2, and UDP3 fractions, as the final digests. Furthermore, the reducing yield proportions of Δ units for the UDP4 and UDP5 substrates demonstrated that Aly2 prefers to produce Δ products when digesting the smallest substrates (UDP4).

**FIGURE 10:**
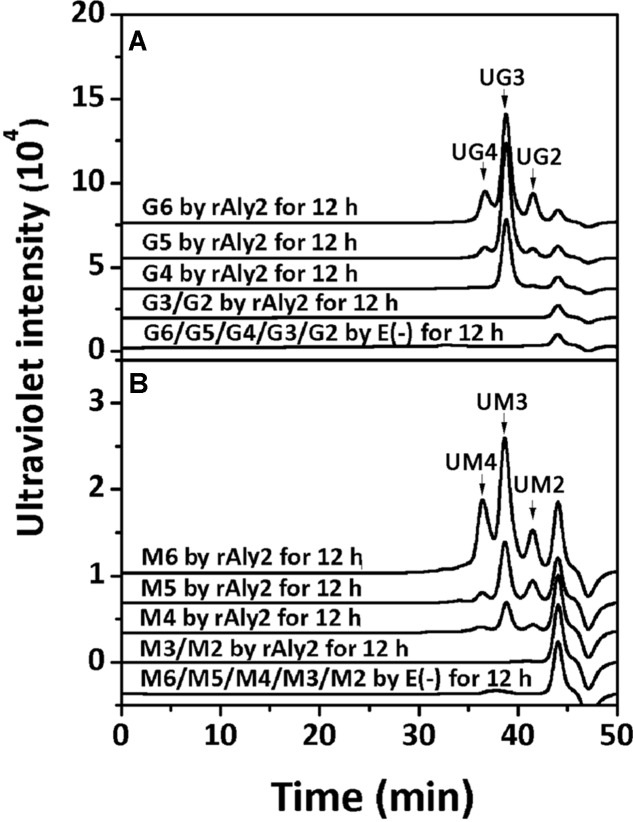
Degradation patterns of rAly2 toward various size-defined saturated oligosaccharide chains enriched with M or G. Degradation of 20 μg saturated oligosaccharide chains including G2 to G6 **(A)** and M2 to M6 **(B)** by rAly2 at optimal condition for 12 h. E (–), controlling group without the enzyme. HPLC analyses were performed using a Superdex^TM^ peptide 10/300 GL column monitored at 235 nm. G6, hexasaccharide of guluronate; M2, disaccharide of mannuronate.

To determine the degradation pattern of the two enzymes toward saturated oligosaccharides substrates, various size-defined saturated oligosaccharide fractions, i.e., G2 to G6 (**Figure 10A**) and M2 to M6 (**Figure 10B**) chains, were reacted with rAly2 for 12 h, using a procedure similar to that for the UDP4 fractions. As shown in **Figure 10**, the enzyme rAly2 degraded G-enriched and M-enriched oligosaccharide substrates that were greater than tetrasaccharide fractions in size. When degrading G6, rAly2 yielded products of saturated trisaccharide (G3) and UG3 or G2 and UG4 fractions. When digesting G5, the enzyme mainly produced saturated G-enriched disaccharide (G2) and unsaturated G-enriched trisaccharide (UG3) and a small quantity of G and UG4, and when reacted with G4, it mainly produced G and UG3 and a small quantity of G2 and UG2 products. The results suggested that during alginate degradation, rAly2 produce a series of small size-defined saturated oligosaccharide products, i.e., G, G2, and G3 from the non-reducing ends of single or different saturated G-enriched oligosaccharide chains.

When reacted with saturated M2 to M6 fractions, the enzyme rAly2 showed a chromatographic profile similar to that with saturated G-enriched oligosaccharide chains. The results indicated that during alginate degradation, rAly2 degrades different saturated M-enriched oligosaccharide substrates to produce various small size-defined saturated oligosaccharide products, i.e., M, M2, and M3 chains from the non-reducing ends of single or different saturated M-enriched oligosaccharide substrates.

## Discussion

Reported bacterial strains of the *Flammeovirga* genus can efficiently degrade and grow on multiple complex polysaccharides, including agarose, alginate, and starch, indicating that they are abundant resources of various efficient polysaccharide-degrading enzymes (Hosoya and Yokota, 2007; Han et al., 2012; Xu et al., 2012). Therefore, many *Flammeovirga* strains have been genome-sequenced and annotated for gene mining and enzyme exploration (Chan et al., 2015; Liu et al., 2015; Dong et al., 2017; Gao et al., 2017). However, so far, only a G-specific (Aly5) and two bifunctional alginate lyases (Aly1 and FsAlyA; Han et al., 2015; Cheng et al., 2017; Zhu et al., 2017) from this genus have been studied. These enzymes were all demonstrated to be endolytic enzymes belonging to the PL7 superfamily. In alginate degradation, the bifunctional enzyme FsAlyA showed similar biochemical characteristics and action modes (e.g., an endolytic substrate-degrading pattern and the molecular size ranges of oligosaccharide products) but a different substrate preference to the G lyase Aly5, even though they shared 78% sequence identity (Zhu et al., 2017). The recombinant bifunctional endolytic enzyme rAly1 from *Flammeovirga* sp. strain MY04 showed variable action modes similar to the natural bifunctional endolytic alginate lyase purified from *Alteromonas* sp. strain No. 272 (Iwamoto et al., 2001; Cheng et al., 2017). They were both efficient at producing monosaccharides, albeit in different yield proportions. However, the two enzymes produced only saturated monosaccharide products of M and G units and lacked unsaturated monosaccharide products of Δ units, which makes obtaining a better understanding of the variable alginate-degrading modes of alginate lyases more difficult.

In this study, Aly2, which is encoded by *Flammeovirga* sp. strain MY04, has been assigned to the PL7 superfamily because it contains the QIH, RSELR, and YFKVGCYTQ motifs that are conserved in the aligned catalytic modules of Alg2 (**Supplementary Figure S1**). Together with Aly1 and three hypothetical alginate lyases from other *Flammeovirga* strains, they form a new subclass within the PL7 superfamily (**Figure 1B**). Furthermore, the recombinant enzyme rAly2 and its NCR-truncated protein rT282N showed some similar alginate-degrading properties to Aly1, e.g., (1) showing high activities toward alginate, PG and PM in a reducing order (**Table 2**); (2) yielding unsaturated G-enriched oligosaccharide products with a specific absorbance at 235 nm three- to sevenfold higher than that of M-enriched ones when degrading associated saturated oligosaccharide chains comparably (**Figure 10**); (3) producing ΔG as the final disaccharide product in alginate degradation (^1^H-NMR spectral analyses, **Figure 8**); and (4) containing ΔG ends in amounts greater than ΔM ends in larger size-defined final oligosaccharide product fractions, e.g., UDP3 fractions (**Figure 8**). Therefore, we deduced that the protein members (including Aly1 and Aly2) belonging to the newly defined subclass of PL7 are G-preferring bifunctional endolytic alginate lyases with variable action modes.

**FIGURE 11:**
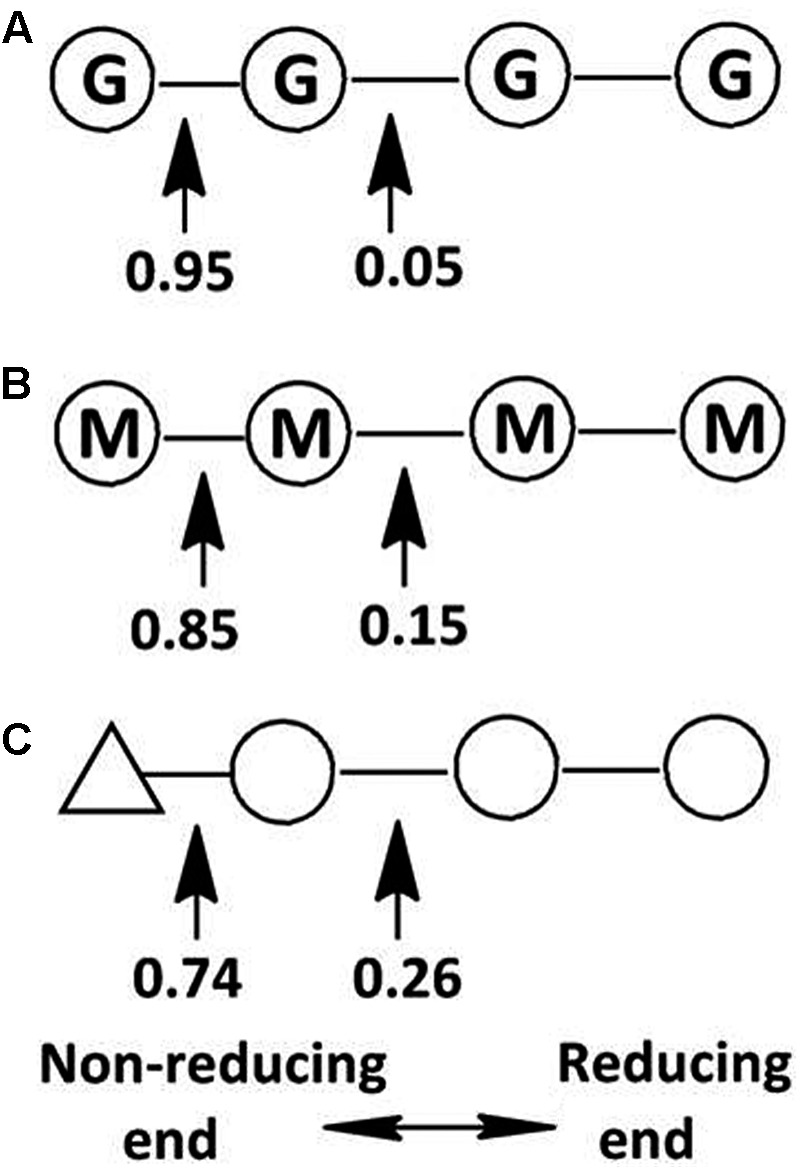
Variable action modes of rAly2 toward tetrasaccharide-sized alginate chains. Arrows indicate possible cleavage sites of rAly2 toward tetrasaccharide chains of G4 **(A)**, M4 **(B)**, and UDP4 **(C)**. Molar proportions of possible oligosaccharide metabolic pathways by rAly2 were calculated by area integration of underlined product fractions in gel filtration HPLC results. Δ, unsaturated sugar unit; M, β-D-mannuronate; G, α-L-guluronate.

This hypothesis has been partially proven in our further observation of the special properties of Aly2. When digesting saturated oligosaccharide chains, e.g., M4, M5, G4, and G5 fractions, the enzyme rAly2 and its NCR-truncated protein rT282N accordingly produced monosaccharide product types of M or G units, accompanied by a yield of various size-defined unsaturated chains, e.g., UM2, UM3, UG2, and UG3 (**Figure 5**), as the final main products. Furthermore, through the chromatographic analyses of the final degradation products of various saturated oligosaccharide chains enriched with M or G, we also demonstrated that rAly2 can produce a series of small size-defined saturated oligosaccharide products from a single or different sugar chains (**Figure 10**), with parallel size enlargements of the oligosaccharide products to substrates. Similar variable substrate-degrading patterns and oligosaccharide-yielding properties were also observed when we used unsaturated oligosaccharide chains as test substrates (**Figure 9**). Furthermore, the M/G contents of the saturated oligosaccharide chains also showed significant effects on the degradation proportions between possible sugar degradation pathways (**Figure 11**), mainly due to the enzyme preference for G over M. Notably, in addition to saturated monosaccharide products, Aly2 also produces unsaturated monosaccharide Δ units, which is a novel property that has not been previously reported for natural or recombinant alginate lyases with variable action modes. Furthermore, our study suggested that Aly2 can produce Δ units when digesting substrates ≥UDP4 fractions instead of smaller size-defined chains, e.g., UDP3 fractions (**Figure 9**). Therefore, we have demonstrated that Aly2 is a G-preferring bifunctional endolytic alginate lyase with variable modes of alginate degradation and the novel capacity to produce every monosaccharide product, including M, G, and Δ units.

## Author Contributions

CP designed the study under the guidance of WH and FL. CP, WH, and FL drafted and corrected the manuscript. CP carried out the experiments. QW prepared UDP3, UDP4, and UDP5 using rAly5. DL prepared polyguluronate and polymannuronate. All authors approved the final manuscript.

## Conflict of Interest Statement

The authors declare that the research was conducted in the absence of any commercial or financial relationships that could be construed as a potential conflict of interest.

## Footnotes

1 http://www.ncbi.nlm.nih.gov

2 http://www.cbs.dtu.dk/services/

3 http://swissmodel.expasy.org/

4 https://en.wikipedia.org/wiki/Simple_Modular_Architecture_Research_Tool

5 http://www.cazy.org/

## Supplementary Material

**FIGURE S1 |** Protein sequence alignment of Aly2 and elucidated PL-7 alginate lyases. Amino acid residues with homologies ≥75% are shaded in black frame and gray back.

**FIGURE S2 |** Non-linear fit curves for the hydrolysis of sodium alginate by rAly2. The initial rates were determined with 0–4 mg/ml of each substrate at 40°C. Error bars represent means of triplicates ± SD.

**FIGURE S3 |** Non-linear fit curves for the hydrolysis of sodium alginate by rT282N. The initial rates were determined with 0–4 mg/ml of each substrate at 40°C. Error bars represent means of triplicates ± SD.

## Abbreviations

Δ: 4-deoxy-L-erythro-5-hexoseulose uronic acid
DP: degree of polymerization
G: guluronate
HPLC: high performance liquid chromatography
M: mannuronate
NMR: nuclear magnetic resonance
PG: polyguluronate
PL7 family: polysaccharide lyase family 7
PM: polymannuronate
SDS-PAGE: sodium dodecyl sulfate polyacrylamide gel electrophoresis
UDP2: unsaturated disaccharide
UDP3: unsaturated trisaccharide
UDP4: unsaturated tetrasaccharide
