# Supplementary figures and images for: A Novel Bifunctional Endolytic Alginate Lyase with Variable Alginate-Degrading Modes and Versatile Monosaccharide-Producing Properties
Source: Front Microbiol. 2018 Feb 8;9:167. doi: 10.3389/fmicb.2018.00167 (PMC5809466; doi:10.3389/fmicb.2018.00167)

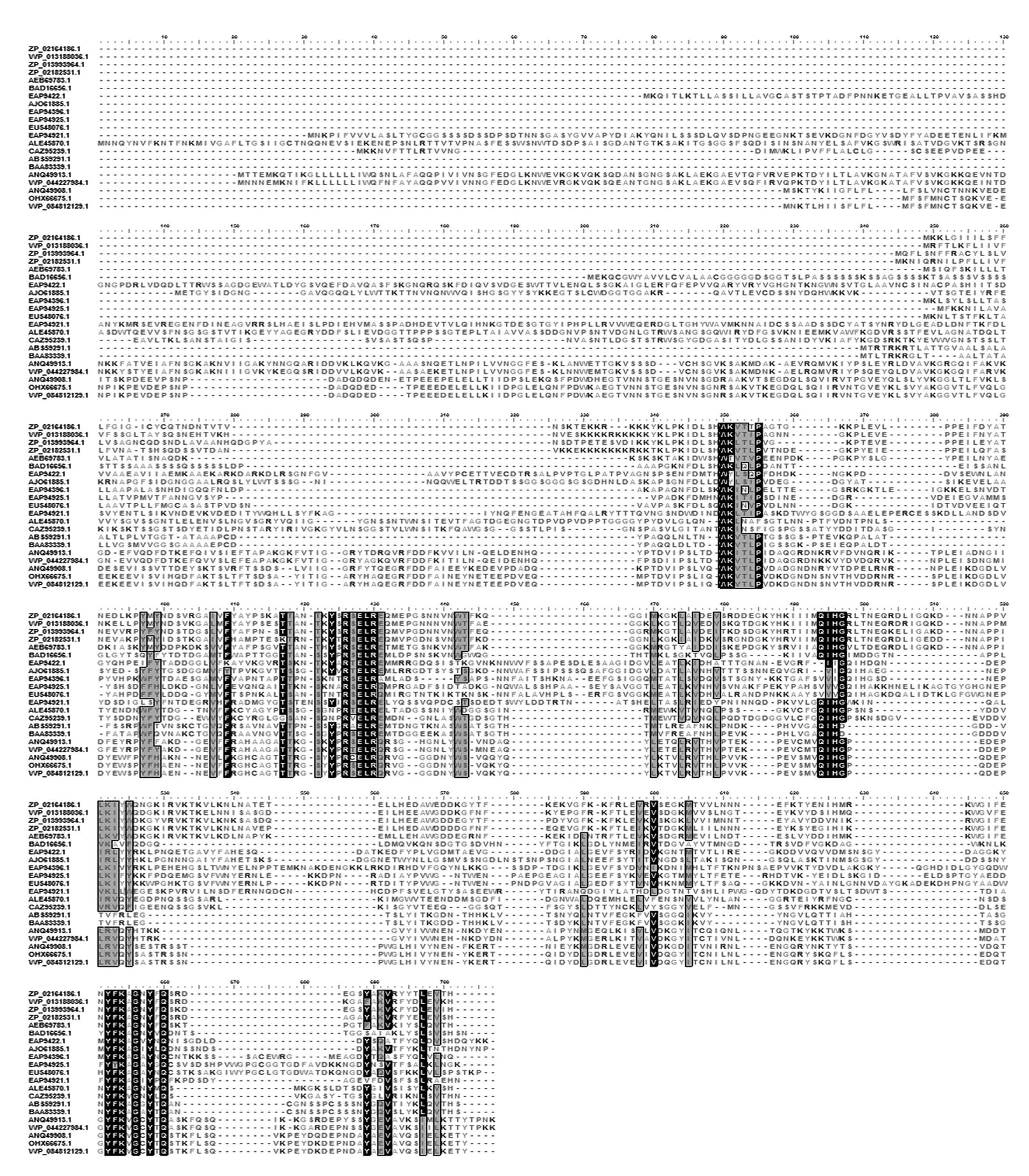

Supplement: Supplementary file 1 [file Image_1.TIF]

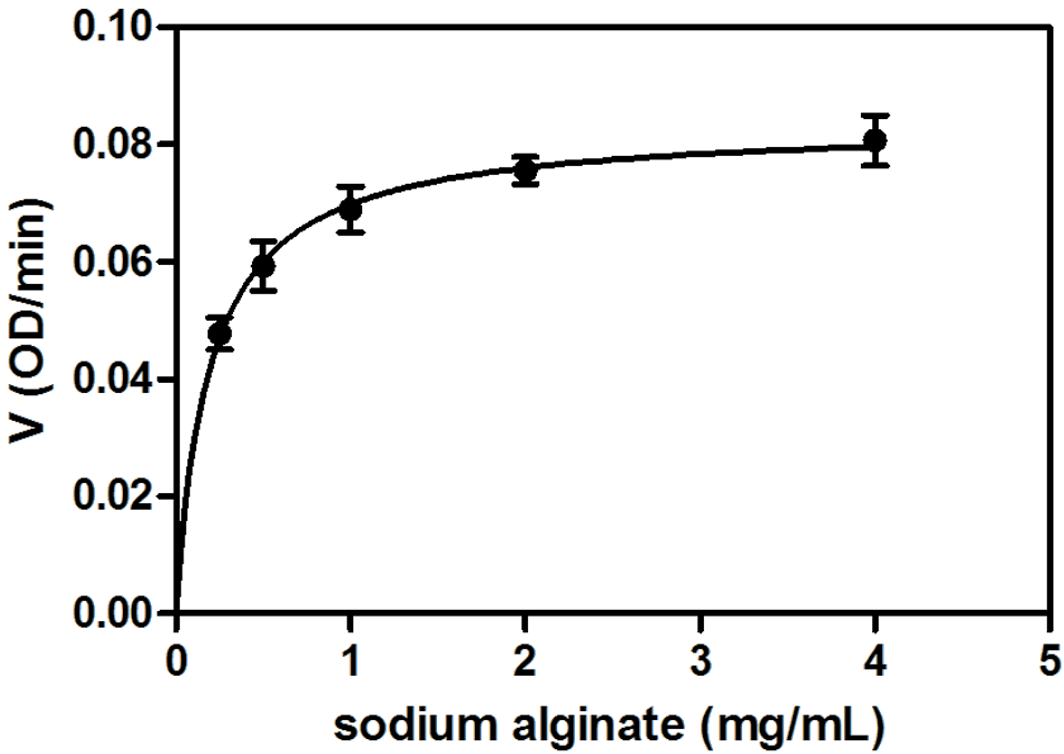

Supplement: Supplementary file 2 [file Image_2.TIF]

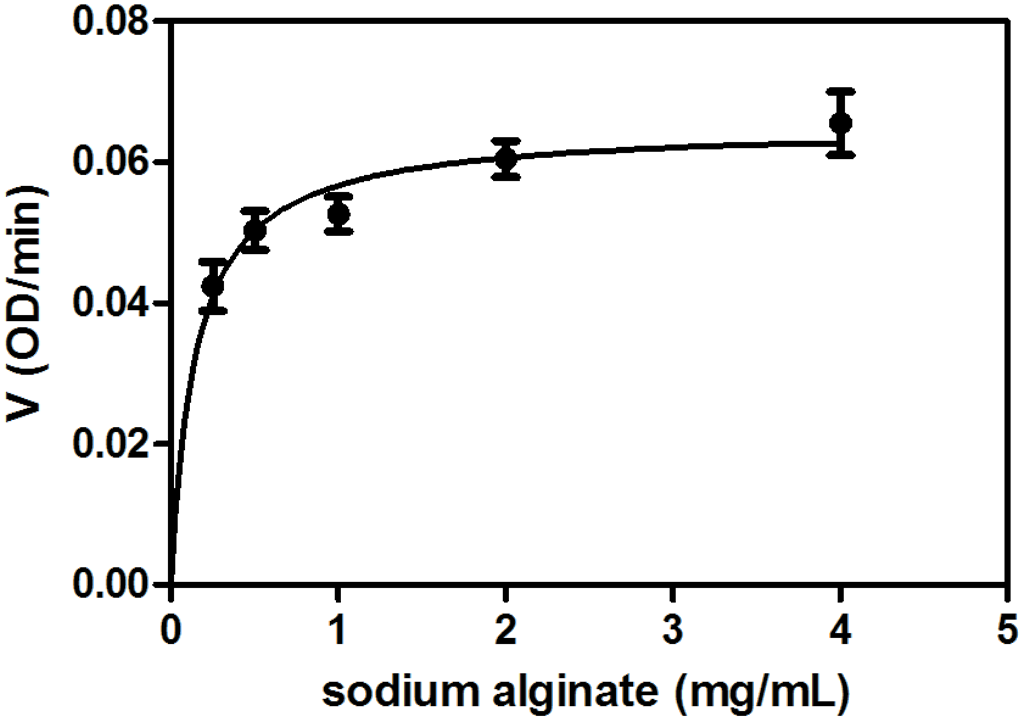

Supplement: Supplementary file 3 [file Image_3.TIF]
